# Fucose Ameliorates *Tritrichomonas* sp.-Associated Illness in Antibiotic-Treated *Muc2^−/−^* Mice

**DOI:** 10.3390/ijms221910699

**Published:** 2021-10-02

**Authors:** Kseniya M. Achasova, Elena N. Kozhevnikova, Mariya A. Borisova, Ekaterina A. Litvinova

**Affiliations:** 1 Siberian Federal Scientific Centre of Agro-BioTechnologies of the Russian Academy of Sciences, 630501 Krasnoobsk, Russia; achasovaks707@gmail.com (K.M.A.); kozhevnikovaen@physiol.ru (E.N.K.); 2 Scientific Research Institute of Neurosciences and Medicine, 630117 Novosibirsk, Russia; 3 Institute of Molecular and Cellular Biology, The Siberian Branch of the Russian Academy of Sciences, 630090 Novosibirsk, Russia; 4 The Laboratory of Biotechnology, Novosibirsk State Agrarian University, 630039 Novosibirsk, Russia; 5 Federal Research Center Institute of Cytology and Genetics of the Siberian Branch of the Russian Academy of Sciences, 630090 Novosibirsk, Russia; mariazolot@yandex.ru

**Keywords:** fucose, protozoa, antibiotic, Mucin2, inflammation, microbiome

## Abstract

The mucus layer in the intestine plays a critical role in regulation of host–microbe interactions and maintaining homeostasis. Disruptions of the mucus layer due to genetic, environmental, or immune factors may lead to inflammatory bowel diseases (IBD). IBD frequently are accompanied with infections, and therefore are treated with antibiotics. Hence, it is important to evaluate risks of antibiotic treatment in individuals with vulnerable gut barrier and chronic inflammation. Mice with a knockout of the *Muc2* gene, encoding the main glycoprotein component of the mucus, demonstrate a close contact of the microbes with the gut epithelium which leads to chronic inflammation resembling IBD. Here we demonstrate that the *Muc2^−/−^* mice harboring a gut protozoan infection *Tritrichomonas* sp. are susceptible to an antibiotic-induced depletion of the bacterial microbiota. Suppression of the protozoan infection with efficient metronidazole dosage or L-fucose administration resulted in amelioration of an illness observed in antibiotic-treated *Muc2^−/−^* mice. Fucose is a monosaccharide presented abundantly in gut glycoproteins, including Mucin2, and is known to be involved in host–microbe interactions, in particular in microbe adhesion. We suppose that further investigation of the role of fucose in protozoan adhesion to host cells may be of great value.

## 1. Introduction

The mammalian gut is inhabited by an enormous number of microorganisms referred to as the gut microbiota. The microbiota is known to contribute to host physiology, particularly the intestinal and systemic immunity [1]. In a healthy gut, the host–microbiota interactions are accurately regulated by the gut epithelial barrier. The barrier is comprised of enterocytes covered with a gel-like mucus layer formed substantially by glycoprotein Mucin2, antimicrobial peptides, and secretory immunoglobulins [2,3]. The barrier prevents close interaction between the gut microbiota and the mucosal immune system, thus precluding excessive immune activation. A breakdown of this barrier leads to a close interaction between the gut microbes and the immune system resulting in the disturbance of the intestinal homeostasis. This might be followed by different pathological conditions, such as IBD, which comprises Crohn’s disease (CD) and ulcerative colitis (UC) [4]. A disruption of the gut barrier may be caused by miscellaneous factors including genetic predisposition, environmental factors (such as nutrition, drug treatment, stress, etc.), status of the immune system and the microbiota [5]. Several genes are identified to be involved in IBD susceptibility including genes of the gut barrier, immune response and microbial defense pathways [6]. In patients with UC, the depletion of mucus thickness [7,8,9] and penetration of bacteria in the inner mucus layer [10] are observed. According to different studies, this may result from the defective differentiation of goblet cells and impaired mucus production [11,12,13,14,15], alteration in mucin glycosylation and sulfation [16,17,18,19] as well as increase of mucin degrading bacteria [20]. The precise etiology of such mucus disruptions is currently unknown and considered to be caused either by genetic factors, or inflammatory response and dysbiosis [14].

In addition to the intestinal barrier dysfunction, IBD are characterized by dysbiosis and concomitant infections. This provides a proinflammatory environment and aggravates inflammation in the gut [21,22]. Hence, one of the most used approaches of IBD treatment is antibiotic therapy. Antibiotics are used to decrease a bacterial load as well as to eliminate a particular infection. However, efficiency of antibiotic therapy is quite controversial and may be followed by several negative side effects [23]. Moreover, some data suggest that antibiotic uptake is a risk factor for IBD onset in childhood [24,25,26]. Studies of the gut microbiome in IBD patients in remission and acute phase demonstrate stronger dysbiosis in the latter [27], suggesting that microbiota may somehow be involved in IBD relapses. Furthermore, treatment of IBD can also be complicated by opportunistic microorganisms widely represented in the gut microflora. A disturbance of the microbiota may result in decrease of colonization resistance for pathogenic and opportunistic microorganisms which can provoke intestinal inflammation [28,29,30]. There are associations between antibiotic treatment and disorders of the gastrointestinal tract caused by opportunistic bacteria [31,32]. Apart from bacteria, the intestinal microbiota comprises fungi, archaea, protists. Less is known about the role of these microorganisms in host physiology and pathogenesis of IBD, but the questions are under investigation [33,34]. Therefore, it is important to understand risks of antibiotic usage, especially in the presence of concomitant infections, for treatment of individuals with defective gut barrier and chronic inflammation.

Animal models are very useful in studying genetically determined defects of the gut barrier and mechanisms of maintaining homeostasis and development of inflammation. *Muc2* gene knockout mice (*Muc2^−/−^*) are widely used as a genetic animal model of IBD. *Muc2^−/−^* mice lack the main secreted glycoprotein Mucin2 forming mucus layer in the gut [35]. As a result, bacteria can directly contact the epithelial cells in the intestine of *Muc2^−/−^* mice [36]. This model is characterized by a chronic gut inflammation that can shift to spontaneous colitis, resembling UC, and colorectal cancer [35,37,38,39]. *Muc2^−/−^* mice are highly susceptible to enteric pathogens colonization [40,41] and the gut barrier disruption [37] displaying severe inflammation, the gut barrier dysfunction and mortality upon such conditions. Moreover, according to our data, antibiotic treatment for eradication of *Helicobacter* spp. infection in Mucin2 deficient mice (*Muc2^−/−^; Kaiso^−/−^* double knockout) did not result in clearance of the pathogen, but led to mortality of the animals [42,43]. Thus, due to the sensitivity to changes in the intestinal environment, Mucin2 knockout model can be used for studying the effects of dysbiosis upon chronic inflammation. In the current investigation, we used *Muc2^−/−^* mice harboring both *Helicobacter* spp. infection and intestinal protozoa *Tritrichomonas* sp. to reveal the effect of antibiotic treatment on the host health.

The microbiota is highly involved in maintaining homeostasis in the gut providing resistance to pathogens [44]. Therefore, development of new approaches for microbiota modulation is of great value, this includes modulation of microbiota during antibiotic therapy. Oligo- and polysaccharides are substances widely investigated as microbiota modulators [45]. In particular, fucose-containing saccharides are under consideration [46]. Fucose is a monosaccharide abundant in the intestinal glycoproteins, including mucin2 [47,48], it is presented in the terminal position of mucin2 polysaccharide chains, thus it is available to bacteria in the gut lumen. Fucose is known to mediate interactions between microbes and host: it serves as a nutrient for microbes [49], can affect virulence of pathogens (demonstrated for *E. coli* [50]), is involved in pathogen adhesion [49,51]. Defective fucosylation in the gut results in alteration of the microbiota [52]. Resident microbiota can stimulate fucosylation in the intestine, thus protecting against inflammation and pathogenic infection [53] Several studies have shown that fucose-containing substances are able to modulate the gut microbiota [54,55,56] and ameliorate antibiotic-induced dysbiosis [57]. Recently we have demonstrated that fucose contributes to amelioration of colitis-associated dysbiosis [58]. Lack of the glycoprotein mucin2 in *Muc2^−/−^* mice is likely result in decrease of the amount of fucose in the intestine. Thus, the protective properties of fucose against infection and inflammation might be reduced in this mouse model. Considering the beneficial effect of fucose on the gut microbiota, in the current study we investigated if supplementation of antibiotic treatment with fucose contributes to the susceptibility to the antibiotic-induced mortality of *Muc2^−/−^* mice.

## 2. Results

### 2.1. Antibiotics-Induced Mortality of Muc2^−/−^ Mice Is Associated with Presence of Tritrichomonas *sp.*

**Figure 1:**
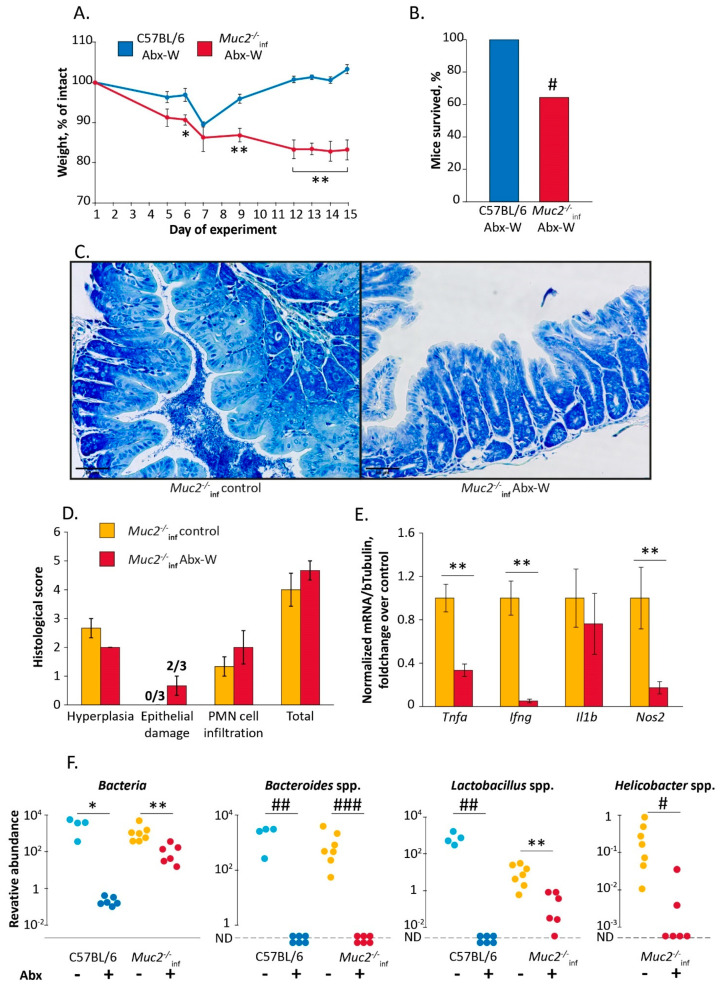
The antibiotic treatment induced mortality of *Muc2^−/−^* mice. (**A**) Body weight dynamics during the antibiotic treatment. *, **—“C57BL/6 Abx-W” vs. “*Muc2^−/−^*_inf_ Abx-W”, *p* < 0.05, *p* < 0.01, Mann–Whitney u-test. (**B**) Survival rate. #—“C57BL/6 Abx-W” vs. “*Muc2^−/−^*_inf_ Abx-W”, *p* < 0.05, Fisher Exact test. (**C**) Azur-II-eosin-stained colonic sections of *Muc2^−/−^* mice (treated with the antibiotics and control group). Scale bar = 50µm. (**D**) Histological score of inflammation in colon of *Muc2^−/−^* mice. (**E**) Gene expression in colon tissue normalized on beta Tubulin (Tubb5) gene. **—differences between groups *p* < 0.01, Mann–Whitney u-test. (**F**) Relative abundance of the gut bacteria in feces on the day 15, normalized on *Mus musculus* 28S rRNA DNA. *, **—differences between groups *p* < 0.05, *p* < 0.01, Mann–Whitney u-test. #, ##, ###—differences between groups *p* < 0.05, *p* < 0.01, *p* < 0.001, Fisher Exact test. ND—not detected. Experimental groups: “Abx-W”—treatment with the antibiotics by adding in drinking water.

First, we tested the effect of the gut microbiota depletion on the health of *Muc2^−/−^* mice. The mice were treated with a cocktail of broad-spectrum antibiotics (clarithromycin, amoxicillin and metronidazole) in drinking water. According to our data, this treatment eradicates *Helicobacter* spp. in C57BL/6 mice [42]. The treatment with the antibiotics resulted in death of 35% of *Muc2^−/−^* mice harboring *Helicobacter* spp. (Hspp) (9 of 14 mice survived, group “*Muc2^−/−^*_inf_ Abx-W”). The same treatment did not affect C57BL/6 mice free of infections (12 of 12 mice survived, group “C57BL/6 Abx-W”) (Fisher Exact test vs. “*Muc2^−/−^*_inf_ Abx-W”: *p* < 0.05) (Figure 1B). We observed significant changes in body weight during the experiment in both “C57BL/6 Abx-W” and “*Muc2^−/−^*_inf_ Abx-W” groups (Friedman chi-squared 46.6 and 21.6, df = 7, *p* < 0.001 and *p* < 0.01 respectively). The body weight loss in “*Muc2^−/−^*_inf_ Abx-W” was greater than in “C57BL/6 Abx-W” on the days 6, 9, and 12–15 (Mann–Whitney u-test: Z = 2.31, *p* < 0.05; Z = 2.94; Z = 3.36; Z = 3.24; Z = 3.1, *p* < 0.01 respectively) (Figure 1A). After the two-week antibiotic treatment, survived *Muc2^−/−^* mice demonstrated severe weight loss compared to C57BL/6, which recovered by the end of the experiment (Figure 1A). To reveal if the mortality of mutant mice was associated with increased inflammation severity, we performed histological and gene expression analysis in colon. We did not observe significant effects of the antibiotic treatment on histological scores; however, 2 of 3 *Muc2^−/−^* mice demonstrated epithelial damage indicated by intensified desquamation (Figure 1C). The antibiotic treatment resulted in decreased expression of proinflammatory genes *Tnfa*, *Ifng* and *Nos2* (Mann–Whitney u-test compared to control group: Z = 3.06; Z = 3.18; Z = 2.72; *p* < 0.01 respectively) and did not affect *Il1b* (Figure 1E) Thus, the antibiotic-induced mortality was not associated with aggravation of inflammation.

*Muc2^−/−^* mice used in the experiment harbored *Helicobacter* spp. infection, so we suggested that the pathogen might be implicated in the mortality of mutant mice. Next, we analyzed fecal microbiota by real-time PCR. The antibiotics decreased *Helicobacter* spp. infection in *Muc2^−/−^* mice (Fisher Exact test: *p* < 0.05, Figure 1F). As expected, the antibiotic treatment resulted in decrease of total gut bacteria determined by measuring the amount of bacterial 16S rRNA gene in feces of *Muc2^−/−^* and C57BL/6 mice (effect of the experimental group by Kruskal–Wallis test: H (3,23) = 18.64, *p* < 0.001, Figure 1F). Gut symbiotic bacteria *Bacteroides* spp. were undetectable after the treatment in mice of both genotypes (Fisher Exact test: *p* < 0.01 for C57BL/6 and *p* < 0.001 for *Muc2^−/−^*_inf_, Figure 1F). *Lactobacillus* spp. also decreased after the antibiotic treatment in *Muc2^−/−^* mice (Mann–Whitney u-test: Z = 2.64, *p* < 0.01, Figure 1F) and were undetectable in C57BL/6 (Fisher Exact test: *p* < 0.01, Figure 1F). Thus, we supposed that the mortality observed was not caused by *Helicobacter* spp. infection, but depletion of the intestinal microbiota was critical for viability of *Muc2^−/−^* mice.

**Figure 2:**
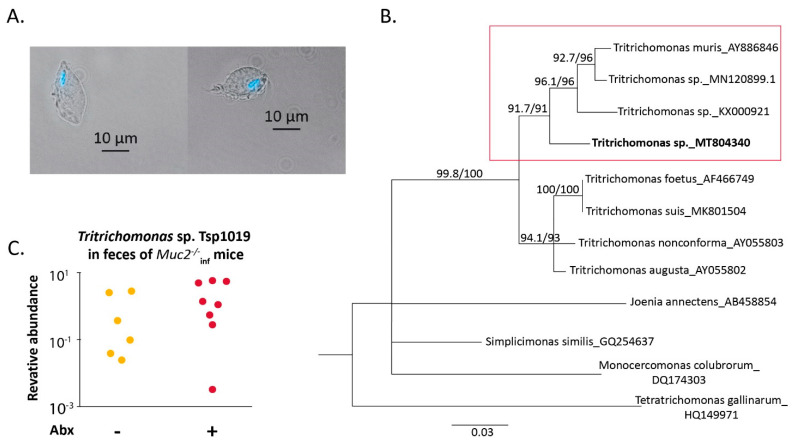
Protozoan microorganism *Tritrichomonas* sp. clone Tsp1019 was detected in the intestine of *Muc2^−/−^* mice. (**A**) Micrograph demonstrates microorganism purified from cecal content stained with Hoechst 33258 (magnification ×400), the bright field and Hoechst 33258 are merged. Scale bar = 10 µm. (**B**) Phylogenetic analysis of 18S rRNA DNA sequence obtained from gut content of *Muc2^−/−^* mice. (**C**) Relative abundance of *Tritrichomonas* sp. Tsp1019 DNA in feces of *Muc2^−/−^* mice on the day 15, normalized on *Mus musculus* 28S rRNA DNA.

Interestingly, microscopic investigation of intestinal contents of *Muc2^−/−^* mice revealed a protozoan microorganism that morphologically resembled *Tritrichomonas* species (Figure 2A). To determine the microorganism, we performed sequencing of 18S rRNA gene and phylogenetic analysis as described previously [59]. We found that the detected protozoan clone Tsp1019 (Tsp) (deposed in GenBank, accession number MT804340) was phylogenetically related to *Tritrichomonas* species found in the gut of rodents (*T. muris* clone 1-6 AY886846, *Tritrichomonas* sp. strain LL5 MN120899.1 and *Tritrichomonas* sp. MEG-2016a KX000921—referred to as *T. musculis*, Figure 2B). Next, using the obtained sequence we designed specific primers for quantitative detection of *Tritrichomonas* sp. clone1019. Importantly, amount of *Tritrichomonas* sp. clone Tsp1019 in feces of *Muc2^−/−^* mice treated with the antibiotics did not differ from level observed in control *Muc2^−/−^* mice (Figure 2C). Thus, the antibiotic treatment did not affect the protozoa, suggesting that the microorganism might be involved in the antibiotic-induced mortality upon bacterial microbiota depletion.

### 2.2. Muc2^−/−^ Mice Free of Infections Recover after Antibiotic Treatment

**Figure 3:**
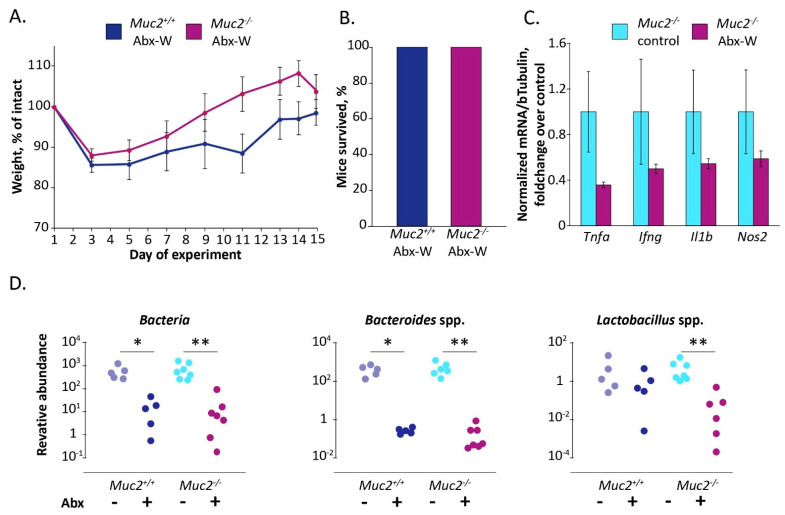
*Muc2^−/−^* mice free of the infections survive after the antibiotic treatment. (**A**) Body weight dynamics upon antibiotic treatment. (**B**) Survival rate. (**C**) Gene expression in colon tissue normalized on beta Tubulin (Tubb5) gene. (**D**) Relative abundance of the gut bacteria in feces on the day 15, normalized on *Mus musculus* 28S rRNA gene. *, **—differences between groups *p* < 0.05, *p* < 0.01, Mann–Whitney u-test. Experimental groups: “Abx-W”—treatment with the antibiotics by adding in drinking water.

To test if the antibiotic-induced death of *Muc2^−/−^* mice resulted from the presence of the protozoan infection, we performed an experiment using *Muc2^−/−^* mice free of infections (including Tsp), *Muc2^+/+^* littermates were used as a control. In contrast to the previous experiment, the antibiotic treatment did not result in severe weight loss and mortality of infection-free *Muc2^−/−^* mice (100% of mice survived) (Figure 3A,B). Moreover, we did not observe significant effects on expression of the proinflammatory genes after the antibiotics administration (Figure 3C). Thus, infection-free mutant mice were less sensitive to antibiotic treatment than infected mice. Therefore, we questioned, if the treatment decreased intestinal bacteria in infection-free mice. In accordance with the previous experiment, the antibiotics decreased the amount of total bacterial 16S rRNA copy number as well as *Bacteroides* and *Lactobacillus* spp. (the effect of the experimental group by Kruskal–Wallis test: H (3,24) = 17.34 for Bacteria, H (3,24) = 17.57 for *Bacteroides* spp., H (3,24) = 13.23 for *Lactobacillus* spp. *p* < 0.001, Figure 3D).

Therefore, the antibiotics did not induce mortality in *Muc2^−/−^* mice free of infections. We assumed that the lethal effect indeed resulted from the presence of Tsp infection upon depletion of the gut microbiota. Further we tested if suppression of the infection contributes to the amelioration of the antibiotic-induced lethal effect in *Muc2^−/−^* mice.

### 2.3. Elimination of Tritrichomonas *sp.* upon Antibiotic Treatment Results in Recovery of Infected Muc2^−/−^ Mice

**Figure 4:**
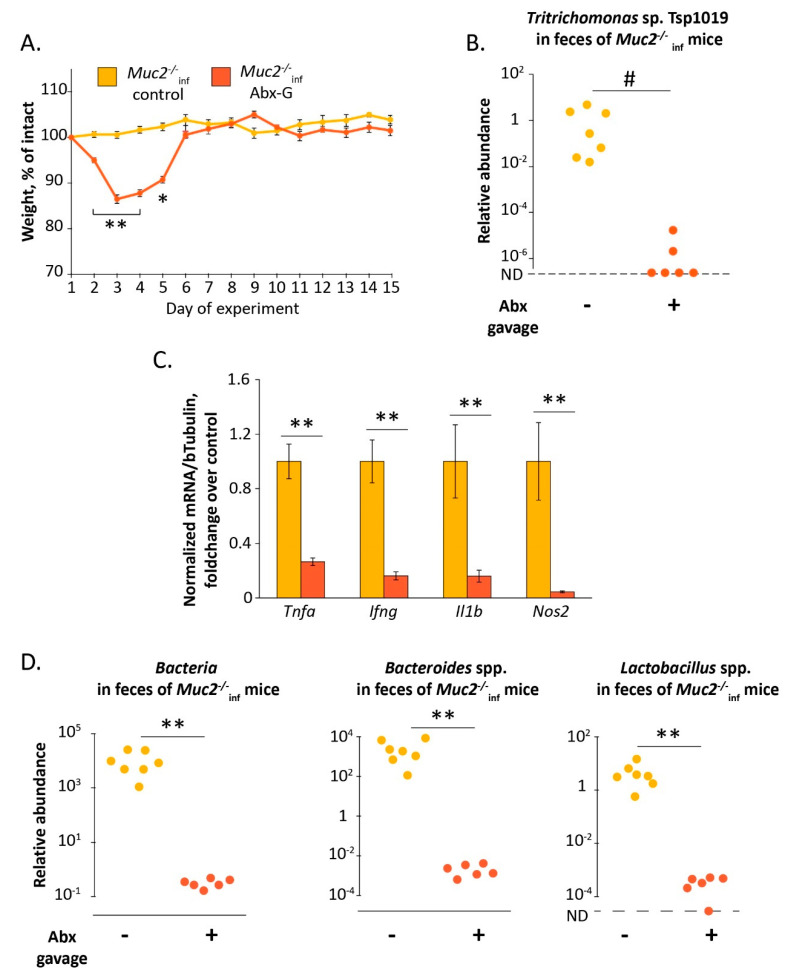
Suppression of *Tritrichomonas* sp. by antibiotic treatment by gavage results in the recovery of the infected *Muc2^−/−^* mice. (**A**) Body weight dynamics upon antibiotic treatment. *, **—“*Muc2^−/−^*_inf_ control” vs. “*Muc2^−/−^*_inf_ Abx-G”, *p* < 0.05, *p* < 0.01, Mann–Whitney u-test. (**B**) Relative abundance of *Tritrichomonas* sp. T.sp1019 in feces on the day 15, normalized on *Mus musculus* 28S rRNA gene. #—differences between groups *p* < 0.05, Fisher Exact test. ND—not detected. (**C**) Gene expression in colon tissue normalized on beta Tubulin (Tubb5) gene. **—differences between groups *p* < 0.01, Mann–Whitney u-test. (**D**) Relative abundance of the gut bacteria in feces on the day 15, normalized on *Mus musculus* 28S rRNA gene. **—differences between groups *p* < 0.01, Mann–Whitney u-test. ND—not detected. Experimental groups: “control”—gavage with drinking water; “Abx-G”—gavage with the antibiotics.

Furthermore, to suppress Tsp infection upon the gut microbiota depletion in *Muc2^−/−^* mice, we treated the mice with the antibiotics by intragastric gavage for two weeks (group “*Muc2^−/−^*_inf_ Abx-G”). According to several studies, this way of antibiotic treatment is effective and has less adverse effects [60,61]. The daily doses of the antibiotics used were the same as those being used during treatment via drinking water in our previous experiments. The dose of metronidazole used was proportionate to those reported to be effective for suppression of *Tritrichomonas* spp. infections [62]. We assumed that treatment by gavage may be of more advantage due to administration of the daily dose in a one-time manner. Indeed, Tsp infection significantly decreased in the group “*Muc2^−/−^*_inf_ Abx-G” compared to the control group “*Muc2^−/−^*_inf_ Cont” (gavaged with drinking water) after two weeks of the treatment (Fisher Exact test: *p* < 0.05, Figure 4B). We observed a significant effect on body weight during the experiment in both groups (Friedman chi-squared 52.7 and 62.8 df = 14, *p* < 0.001 respectively for “*Muc2^−/−^*_inf_ Cont” and “*Muc2^−/−^*_inf_ Abx-G” groups, Figure 4A). The antibiotic treatment resulted in significant decrease of body weight in *Muc2^−/−^* mice in comparison to the control mice. The body weight loss in “*Muc2^−/−^*_inf_ Abx-G” group was greater than in “*Muc2^−/−^*_inf_ Cont” from the day 2 to the day 5 (Mann–Whitney u-test: Z = 3, *p* < 0.01; Z = 2, *p* < 0.05), but by the day 6 the mice recovered (Figure 4A). Similar to the previous experiments antibiotic treatment via gavage caused decrease of total bacteria, *Bacteroides* spp. and *Lactobacillus* spp. (Mann–Whitney u-test: Z = 2.08; *p* < 0.01; Figure 4D). Thus, a suppression of Tsp upon an alteration of the gut microbiota was associated with the recovery of the mutant mice after the antibiotic treatment. Moreover, in accordance with the fist experiment (Figure 1E) the treatment of the infected mice with the antibiotics resulted in decreased expression of the proinflammatory genes *Tnfa*, *Nos2*, *Ifng*, *Il1b*, (Mann–Whitney u-test: Z = 2.92; Z = 2.64; Z = 2.78; *p* < 0.01; Figure 4C). Therefore, we assumed that the antibiotic-induced death of the *Muc2^−/−^* mice observed previously resulted from insufficient suppression of Tsp infection upon the bacterial microbiota depletion. Next, we attempted to improve antibiotic-induced mortality by supplementation with the monosaccharide L-fucose.

### 2.4. L-Fucose Prevents an Expansion of Tritrichomonas *sp.* and Ameliorates Antibiotic-Induced Emaciation in Infected Muc2^−/−^ Mice

**Figure 5:**
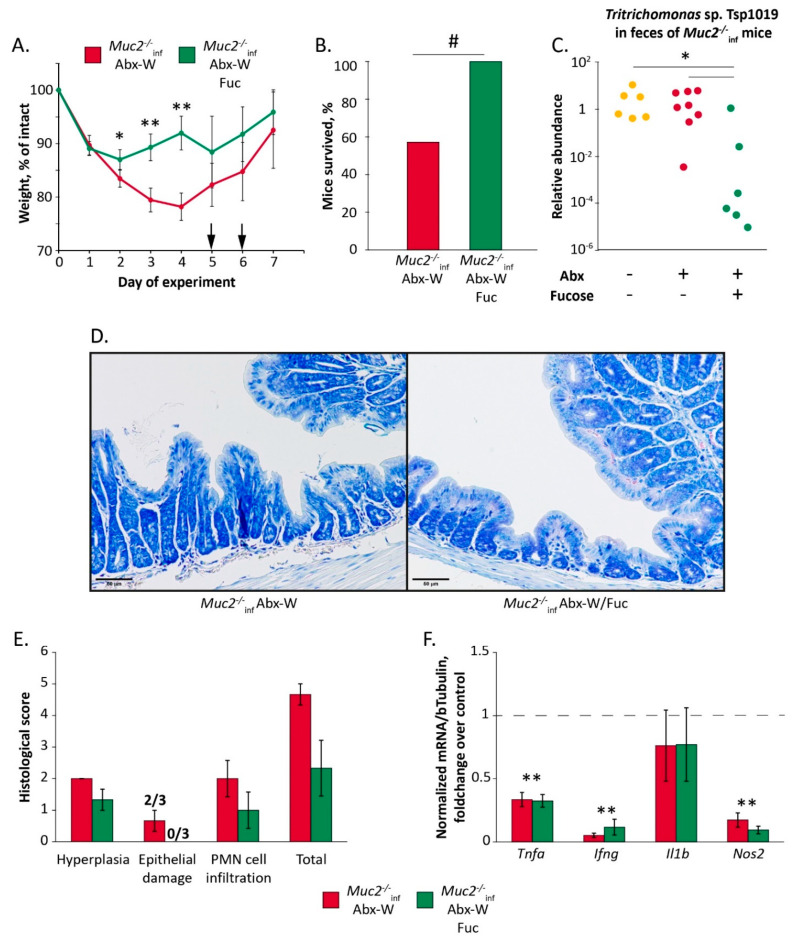
L-fucose suppresses *Tritrichomonas* sp. and ameliorates antibiotic-induced emaciation in infected *Muc2^−/−^* mice. (**A**) Body weight dynamics upon treatment with antibiotics and L-fucose. *, **—*p* < 0.05 and *p* < 0.01, Mann–Whitney u-test. Arrows indicate the days when death was observed. (**B**) Survival rate. #—*p* < 0.05, Fisher Exact test. (**C**) Relative abundance of *Tritrichomonas* sp. in feces normalized on *Mus musculus* 28S rRNA DNA. *—differences between groups *p* < 0.05, Mann–Whitney u-test. (**D**) Azur-II-eosin-stained colonic sections of *Muc2^−/−^* mice (treated with the antibiotics and control group). Scale bar = 50µm. (**E**) Histological score of inflammation in colon of *Muc2^−/−^* mice. (**F**) Gene expression in colon tissue normalized on beta Tubulin (Tubb5) gene. **—differences compared to mRNA level in control group indicated by dotted line, *p* < 0.01, Mann–Whitney u-test. Experimental groups: “Abx-W”—treatment with the antibiotics via drinking water for 7 days; “Abx-W/Fuc”—treatment with antibiotics with L-fucose via drinking water for 7 days.

In this experiment, the mice were treated with the antibiotics via drinking water in dosage same to the previous experiments (“*Muc2^−/−^*_inf_ Abx-W” group) or with the antibiotics supplemented with 0.1% L-fucose (“*Muc2^−/−^*_inf_ Abx-W/Fuc” group) for 7 days. Similar to the previous experiments, the antibiotic treatment resulted in a severe weight loss and death of the mutant mice (8 of 14 mice survived in “*Muc2^−/−^*_inf_ Abx-W” group). The mortality of the mice was observed on the days 5 and 6 (indicated with arrows in Figure 5A). Interestingly, supplementation of the antibiotic treatment with L-fucose resulted in 100% survival of the *Muc2^−/−^* mice (12 of 12 mice). The difference in survival rate was of significance with *p* < 0.05 (Fisher Exact test, Figure 5B). The both treatments significantly affected body weight (Friedman chi-squared 13.7, df = 6, *p* < 0.05 for “*Muc2^−/−^*_inf_ Abx-W” group and 18.4, df = 6, *p* < 0.01 for “*Muc2^−/−^*_inf_ Abx-W/Fuc” group). However, the body weight loss in “*Muc2^−/−^*_inf_ Abx-W” was greater than in “*Muc2^−/−^*_inf_ Abx-W/Fuc” on the days 2, 3 and 4 (Mann–Whitney u-test: Z = 2.05, *p* < 0.05; Z = 3.18; Z = 3.13, *p* < 0.01 respectively) (Figure 5A).

In accordance with the previous results, the antibiotics provided in drinking water did not affect relative abundance of the Tsp in feces, but supplementation of the treatment with L-fucose resulted in decrease of Tsp DNA in 13 times (Mann–Whitney u-test: Z = 2.5 for “*Muc2^−/−^*_inf_ Abx-W/Fuc” vs. “*Muc2^−/−^*_inf_ Abx-W” *p* < 0.05, Figure 5C). Thus, recovery of *Muc2^−/−^* mice after the antibiotic treatment was associated with suppression of Tsp. infection by L-fucose.

In the previous experiment we did not observe significant histological changes in colon of *Muc2^−/−^* mice after the antibiotics, with the exception of epithelial damage scores that had tended to increase (Figure 1D). Supplementation of the antibiotics with L-fucose did not induce significant changes in colon histology, but abrogated slightly increased epithelial damage score (Figure 5D,E). The antibiotic treatment resulted in a decrease of proinflammatory *Tnf*, *Ifng* and *Nos2* genes expression (Mann–Whitney u-test compared to control group indicated by dotted line: Z = 3.06; Z = 3.18; Z = 2.72; *p* < 0.01 respectively Figure 5F) and L-fucose did not ameliorate the mRNA level of *Tnf*, *Ifng*, and *Nos2* genes (Mann–Whitney test over control group indicated by dotted line: Z = 2.93, Z = 2.78; Z = 2.78; *p* < 0.01 respectively Figure 5F). Therefore, recovery of the mice and suppression of Tsp. infection were not accompanied with amelioration of the immune suppression.

**Figure 6:**
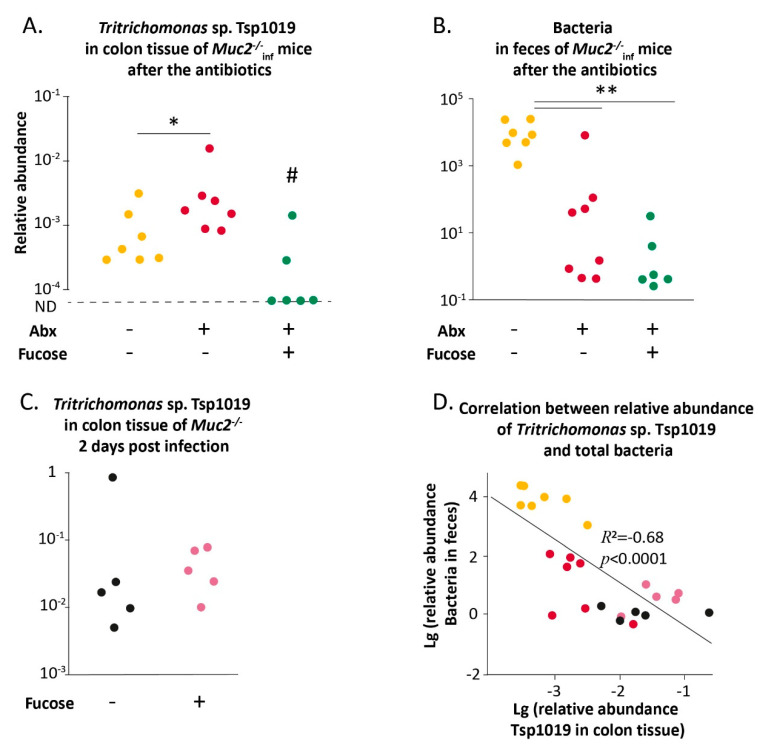
L-fucose prevents an expansion of *Tritrichomonas* sp. in colon of *Muc2^−/−^* mice upon antibiotic-induced microbiota depletion. (**A**) Relative abundance of *Tritrichomonas* sp. in colon tissue of *Muc2^−/−^* mice after treatment with antibiotics and antibiotics supplemented with L-fucose, normalized on *Mus musculus* 28S rRNA DNA. *—differences between groups *p* < 0.05, Mann–Whitney u-test. #—*p* < 0.05, Fisher Exact test. ND—not detected. (**B**) Relative abundance of the gut bacteria in feces of *Muc2^−/−^* mice after treatment with antibiotics and antibiotics supplemented with L-fucose normalized on *Mus musculus* 28S rRNA gene. **—differences between groups *p* < 0.01, Mann–Whitney u-test. (**C**) Relative abundance of *Tritrichomonas* sp. in colon tissue of *Muc2^−/−^* mice after infection via gavage. (**D**) Spearman correlation between relative abundance of Tsp1019 in colon tissue and bacteria in feces.

*Tritrichomonas* species attach to the epithelial cells, which is critical for establishment of the infection [63]. In mice lacking mucin2 intestinal epithelial cells are more vulnerable to contact with microbes, so next we assessed the amount of Tsp DNA in the colon tissue of *Muc*2^−/−^ mice. Remarkably, treatment with the antibiotics resulted in an increase of Tsp in the colon tissue (“*Muc2^−/−^*_inf_ control” vs. “*Muc2^−/−^*_inf_ Abx-W” Mann–Whitney u-test: Z = 2.04 *p* < 0.05, Figure 6A). L-fucose promoted decrease of the microorganism in the colon tissue, Tsp DNA was detected only in 2 of 6 mice (Fisher Exact test: *p* < 0.05 for “*Muc2^−/−^*_inf_ Abx-W/Fuc” vs. “*Muc2^−/−^*_inf_ control” and “*Muc2^−/−^*_inf_ Abx-W/Fuc” vs. “*Muc2^−/−^*_inf_ Abx-W”, Figure 6A). In accordance with the previous results, antibiotics caused decrease of fecal bacteria (Mann–Whitney u-test: Z = 2.83, *p* < 0.01, Figure 6B). We assumed that bacterial microbiota depletion might contribute to Tsp. expansion and L-fucose might provide partial microbiota reconstitution, thus promoting recovery of *Muc2^−/−^* mice after the antibiotic treatment. However, L-fucose did not affect the amount of total bacterial DNA after the antibiotics in *Muc2^−/−^* mice (Mann–Whitney u-test: Z = 2.93, *p* < 0.01, Figure 6B).

Therefore, L-fucose either interfered with the attachment of Tsp to colon cells or negatively affected the viability of Tsp in the intestinal lumen. To test this, *Muc2^−/−^* mice with intact microbiota were infected with Tsp. via gavage of fecal suspension containing *Tritrichomonas* sp. The mice were provided with drinking water supplemented with L-fucose for two days post-inoculation and then were tested for Tsp. DNA in colon tissue. The gavage resulted in infection with *Tritrichomonas* sp. that was detected in all inoculated mice. Supplementation with L-fucose did not affect amount of Tsp. DNA in colon tissue (Figure 6C). Thus, L-fucose did not alter the viability of *Tritrichomonas* sp. in the intestine. It should be noted that the mice infected with *Tritrichomonas* sp. (Figure 6C) did not demonstrate deterioration of well-being despite the detected level of Tsp in intestinal tissue being higher to that observed after the antibiotics (Figure 6B) (Mann–Whitney u-test: Z = 3.07, *p* < 0.01). Therefore, intact bacterial microbiota seemed to be crucial factor for outcomes of Tsp. infection in *Muc2^−/−^* mice. In fact, we observed negative correlation between bacteria in feces and Tsp. in colon tissue (Spearman correlation: *R* = −0.68, *p* < 0.0001, Figure 6D). Thus, normal bacterial microbiota might affect colonization of *Tritrichomonas* sp. and upon depletion of the microbiota L-fucose is able to protect from expansion of Tsp in the colon.

## 3. Discussion

Here we investigated the effect of the gut microbiota and the presence of the infections on susceptibility to antibiotic treatment in a host with a disturbed gut barrier. We made use of *Muc2^−/−^* mice lacking main intestinal secreted mucin, so the gut microbiota closely contacts the intestinal epithelia and the immune cells [36]. Previously, we have shown that *Muc2^−/−^Kaiso^−/−^* double knockout mice are extremely sensitive to antibiotics used for eradication of *Helicobacter* spp. infection. The treatment not only was ineffective for elimination of the pathogen, but also resulted in mortality of the animals [42,43]. Moreover, Tadesse and colleagues mentioned the rapid death of *Muc2^−/−^* mice upon antibiotic treatment in the discussion [64], but to our knowledge, the data have not been published. In the current study we demonstrated that antibiotics caused dramatic weight loss (Figure 1A) and mortality (Figure 1B) in *Muc2^−/−^* mice, which was accompanied by depletion of the symbiotic microbiota as well as a decrease of *Helicobacter* spp. infection (Figure 1F). The interesting finding was that the *Muc2^−/−^* mice also harbored protozoan microorganisms identified as *Tritrichomonas* sp. T.sp1019. Phylogenetic analysis revealed that the microorganisms are related to the common murine protozoans *T. muris* and *T. musculis* (Figure 2B). Moreover, the infection was also detected in the mutant mice after the antibiotic treatment (Figure 2C). *Tritrichomonas* spp. are generally considered to be non-pathogenic commensal microbiota of mice, but monitoring of the microorganisms in SPF-facilities is under discussion [65]. *Tritrichomonas* spp. are known to affect host physiology by changing the gut proteome [66] and triggering immune responses in the intestine [67,68]. In the previous studies, the mice were not tested for presence of protozoan microorganisms [42,43], so we assumed Tsp. probably being involved in the antibiotic-induced mortality of *Muc2^−/−^* mice.

We tested if the antibiotic treatment has such a negative effect on *Muc2^−/−^* mice free of the infections (including Tsp). Turned out that the mice without infections did not demonstrate mortality and recovered by the end of two-week treatment with the antibiotics (Figure 3). Thus, depletion of the gut microbiota by itself did not cause the adverse outcome observed. Then we examined if suppression of *Tritrichomonas* sp. infection upon microbiota depletion ameliorates the antibiotic-induced emaciation in *Muc2^−/−^* mice. We did not have isolated cultures of *Tritrichomonas* sp., we infected mice through intragastric gavage with fecal microbiota from mice positive for *Tritrichomonas* sp. Tsp1019. To avoid the effect of the infection inoculation we used these infected mice to generate offspring being born with the infection. The infected mice were treated with the antibiotics by intragastric gavage, this approach was reported to be effective and less harmful [60,61]. The treatment resulted in a decrease of *Tritrichomonas* sp. in *Muc2^−/−^* mice (Figure 4B), probably due to appropriate concentration of metronidazole in antibiotic cocktail. Tsp. suppression was associated with complete body weight recovery by the 6th day of the treatment (Figure 4A). Thus, we supposed that the antibiotic-associated body weight loss in mice lacking mucin2 resulted from the presence of *Tritrichomonas* sp. in context of the bacterial microbiota depletion.

The gut bacterial microbiota is known to promote resistance to pathogens [44] and to affect outcomes of various protozoan infections [69]. Some symbiotic bacteria seem to have inhibitory effects on several protozoan parasites [70], thus protecting the host organism. During the infection, *Tritrichomonas* and *Trichomonas* spp. attach to the host epithelial cells leading to the barrier dysfunction and cell death [63]. Gut bacteria are known to mediate epithelium turnover and repair. It was shown that antibiotic-treated mice demonstrate decreased turnover of the intestinal epithelial cells [71] and mortality induced by DSS-treatment [72]. Thus, we hypothesized that reconstitution of the bacterial microflora may ameliorate the antibiotic-associated emaciation in *Muc2^−/−^* mice with *Tritrichomonas* sp. infection.

We attempted to modulate the microbiota depletion by supplementing the antibiotic treatment with fucose upon insufficient suppression of *Tritrichomonas* sp. Mucin2 is a heavily O-glycosylated glycoprotein that serves not only as the main component of host protecting mucus, but also as a nutritional niche for commensal bacteria in the gut. Fucose is a terminal monosaccharide of Mucin2 glycoprotein, it contributes to host-microbiome interaction [73]. Moreover, fucose-containing substances are known to modulate the gut microbiota [54,55,56], fucose monosaccharide contributes to amelioration of colitis-associated dysbiosis [58]. Furthermore, it was shown that fucose-containing polysaccharide fucoidan improved antibiotic-induced dysbiosis and promoted the recovery of gut microbiome in mice [57]. Contrary to our assumption, L-fucose did not affect the number of total bacteria in the gut upon the antibiotic treatment (Figure 6B), but it did ameliorate the antibiotic-induced weight loss in the mutant mice (Figure 5A,B). Interestingly, the observed recovery of the mice was associated with suppression of Tsp infection in colon by L-fucose (Figure 6A). At the same time, treatment with the antibiotics did not cause mortality in *Muc2^−/−^* mice free of the infections and 100% of the animals recovered by the end of the experiment (Figure 3). Altogether our results imply that the antibiotic-induced emaciation resulted from the presence of *Tritrichomonas* sp. infection upon the microbiota depletion in the host with a compromised barrier in the gut.

Antibiotic treatment is known to suppress immune function. For instance, broad-spectrum antibiotics impair leukocytes count by suppression of hematopoiesis [74]. We observed inhibition of the proinflammatory genes expression in the colon tissue (Figure 1E). Thus, the immune suppression in mice lacking Mucin2 in combination with increase of *Tritrichomonas* sp. in colon, probably, was critical for the health of mutant mice. L-fucose did not modulate suppressed immune function (Figure 5F). However, L-fucose, as well as intact bacterial microbiota, prevented *Tritrichomonas* sp. expansion, we assume that the monosaccharide or intestinal bacteria affect the infection through modulation of adhesion in mice lacking mucin2. When attached to the epithelial cells, trichomonads become cytotoxic causing cell damage, loss of function and cell death [63]. Thus, *Tritrichomonas* spp. colonization may result in intoxication and alteration of nutrition and energy uptake. Mechanisms of the adhesion are not completely understood, the most explored are *Tritrichomonas foetus* and *Trichomonas vaginalis*. The studies indicate several surface molecules mediating membrane-membrane interactions of the parasites with host epithelia. These include sialic acid-binding lectins, adhesins, lipophosphoglycan (LPG) and cysteine proteases [63,75]. According to our analysis *T. foetus* is phylogenetically close to *Tritrichomonas* sp. T.sp1019, so we suppose that the adhesion mechanisms of the microorganisms are likely to be similar. Saccharides on the parasite and host cells are involved in cell-cell interaction and adhesion. For instance, sialic acid is known to inhibit *T. foetus* adhesion in vitro [76,77] due to presence of sialic acid-binding lectin in the parasite membrane [78]. Thus, *T. foetus* can use sialic acid on the host cells for adhesion. Membrane of *T. foetus* also contains LPG that binds to host lectins promoting the adhesion [79]. Several studies show that LPG of *T. foetus* contains several saccharides with fucose and mannose being prevalent ones [80], but the surface saccharide patterns in different isolates may vary [81]. Thus, it is possible that fucose can be involved in *Tritrichomonas* spp. pathogenesis, but this needs further investigations. Yet, it was shown that L-fucose inhibits phagocytosis in *T. vaginalis*, probably by blocking mannose receptors on the microorganism [82]. It should be noted that the mechanisms described were explored mostly in vitro, using cells from different hosts, which can differ by glycosylation patterns. Therefore, the effects obtained in one cell culture may not be reproduced in the other. Moreover, to our knowledge, the mechanisms have not been verified in vivo.

Currently, oligosaccharides, including those containing fucose, are considered to be prospective compounds for microbiota modulation, particularly by blocking the microbe adhesion to host cells [83]. Therefore, understanding the mechanisms of protozoa adhesion to the host cells will promote development of new therapeutic approaches for urogenital and gastrointestinal diseases treatment. The human gastrointestinal tract is inhabited by several protozoa, including members of trichomonads—*Dientamoeba fragilis* and *Pentatrichomonas hominis* known to cause gastrointestinal symptoms [84]. Here we demonstrate that treatment with broad-spectrum antibiotics, aimed to eradicate *Helicobacter* spp., led to emaciation and mortality in mice with deficient barrier in the gut due to blooming of *Tritrichomonas* sp. T.sp1019. The effective suppression of the protozoa by the antibiotic gavage and L-fucose supplementation promoted recovery in the mice. Current approaches being used for IBD treatment often do not consider the variety of the microorganisms inhabiting the gastrointestinal tract [85,86], including protozoa. Our results imply that this may result in health deterioration in individuals defective in gut barrier due to overgrowth of non-bacterial agents. Thus, when choosing a therapy, it is important to consider a potential protozoa infection, monitor the microorganisms and, if necessary, make use of drugs for protozoa elimination. One of the successful ways to treat a protozoa infection may be preventing the adhesion to the epithelial cells. Thus, understanding the mechanisms of adhesion and developing drugs affecting these mechanisms are of great perspective.

## 4. Materials and Methods

### 4.1. Mice and Housing Conditions

The study was conducted using the equipment of the Center for Genetic Resources of Laboratory Animals at the Institute of Cytology and Genetics of the Siberian Branch of the Russian Academy of Sciences (ICG SB RAS), supported by the Ministry of Education and Science of the Russian Federation (Unique identifier of the project RFMEFI62117X0015). All experimental procedures were carried out according to Russian legislation in agreement with the Good Laboratory Practice standards (directive #267 from 19.06.2003 of the Ministry of Health of the Russian Federation, Moscow, Russian Federation) and the European Convention for the protection of vertebrate animals used for experimental and other scientific purposes; all procedures were approved by the inter-institutional bioethical committee, protocol #28 (19 June 2015).

Two animal colonies were used in the experiments. The one was positive for *Helicobacter* spp. (Hspp) and negative for all other pathogens listed in the annual FELASA 2014 recommendations [87]. The second was negative for all listed pathogens, further on referred to as mice free of infections. In accordance with the FELASA 2014 recommendations for surveillance, pathogens testing was conducted quarterly in sentinel mice that received both dirty bedding and water from colony mice for a 3-month period. During the experiments protozoan microorganisms were detected in the first colony of *Muc2^−/−^* mice harboring *Helicobacter* spp. The microorganisms were determined as *Tritrichomonas* sp. (Tsp), which is not listed in the annual FELASA 2014 recommendations. In all experiments this microorganism was detected only in *Muc2^−/−^* and *Muc2^+/+^* littermates within “infected” groups.

All animals were housed in single-sex groups of 3-6 mice in individually ventilated caging systems (Optimice^®^, Animal Care Systems, Centennial, CO, USA). The light/dark photoperiod was 14 h/10 h (light off at 16:00 h), the temperature was 22–24 °C, the humidity was 30%–60%, the air exchange was 7–10 volumes per hour. The mice were provided with sterile food (Mouse Maintenance autoclavable, V1534-300, Sniff, Spezialdiäten, GmbH, Spezialdiäten, Germany) and sterile water ad libitum. All experimental groups comprised 8-10-week-old female mice. We used female mice, as male mice obtained from the breeding were used in another investigation. The first experiment was carried out using *Muc2^−/−^* mice backcrossed on C57BL/6JNskrc substrain in our facility and C57BL/6JNskrc mice as a control. *Muc2^−/−^* mice harbored both Tsp and Hspp infections and control C57BL/6JNskrc were free of the infections. All other experiments were conducted using *Muc2^−/−^* and *Muc2^+/+^* littermates harboring Tsp or free of infections.

### 4.2. Tritrichomonas *sp.* Colonization

To obtain mice infected with Tsp., 5 female and 5 male heterozygous (*Muc2^+/−^*) mice free of infections were gavaged intragastrically with fecal suspension from infected animals. To prepare the suspension 3 fecal boli were homogenized in 1 mL of drinking water, filtered through 70 µm cell-strainer (Corning, New York, NY, USA) and diluted up to 1.5 mL. Mice were gavaged with 100 µL of the suspension for three days. In 14 days after infection all mice were positive for Tsp. detected in feces using primers designed specific to *Tritrichomonas* sp. clone Tsp1019 sequence (see below). These infected mice were crossed to obtain infected *Muc2^+/−^* used for subsequent breeding (infection and breeding strategies are pictured in Supplementary Materials Figure S1).

### 4.3. Antibiotic and L-Fucose Treatment

In the study a mix of three antibiotics (clarithromycin, amoxicillin, and metronidazole) was used. Antibiotics concentrations were 0.2 mg/mL for clarithromycin and metronidazole, 0.6 mg/mL for amoxicillin when added in drinking water in accordance with previously used protocol [42]. The dosage for intragastric gavage was 25 mg/kg for clarithromycin and metronidazole, 75 mg/kg for amoxicillin. The dosage of metronidazole was proportionate to that being used for treatment of *Trichomonas* spp. in mice [62]. L-fucose was added in the drinking water in the final concentration of 0.1% as reported previously [58].

### 4.4. Experimental Design

In the first (Figure 1 and Figure 2) and second (Figure 3) experiments mice were treated with the antibiotics by adding in drinking water for 14 days (“Abx-W” groups), control mice obtained the drinking water (“control” groups). During the experiment mice were weighted. Some mutant mice with the infections died on the 13 and 14 days of the experiment. After two-week treatment fecal samples were collected from survived mice and the mice were euthanized using CO_2_.

In the third experiment (Figure 4) mice were treated with the antibiotics by intragastric gavage for 14 days (“Abx-G” group). To estimate if the gavage procedure affected weight loss, control mice were gavaged with drinking water in parallel with the antibiotic treatment (“control” group). During the experiment mice were weighted, after two-week treatment fecal samples were collected and the mice were euthanized using CO_2_.

In the fourth experiment (Figure 5) mice were treated with the antibiotics (“Abx-W” group) and antibiotics supplemented with L-fucose (“Abx-W/Fuc” group) via drinking water for 7 days. Some mice from the “Abx-W” group died on the 5 and 6 days of the experiment. Mice were weighed, fecal samples were collected from survived mice. Mice were euthanized using CO_2_.

In the fifth experiment (Figure 6) *Muc2^−/−^* mice free of infections were inoculated with Tsp infection via gavage. For two days after the inoculation mice were provided with 0.1% L-fucose (“fucose” group) or drinking water (“control” group). Mice were euthanized in two days post-infection using CO_2_.

For microbiota analysis fecal and colon tissue samples were stored at −20 °C until the analysis. Colon tissue samples for gene expression were frozen in liquid nitrogen and stored at −70 °C until the extraction. Colon tissues for histological analysis were fixed in 4% paraformaldehyde.

### 4.5. Fecal and Colon DNA Extraction and Bacteria Real-Time PCR

Fecal and colon tissue DNA was extracted using QIAamp Fast DNA Stool Mini Kit (Qiagen, Hilden, Germany) according to the manufacturer’s instructions. Briefly, samples stored at −20 °C were homogenized and lysed in Inhibitex Buffer at 70 °C for 30 min and centrifuged at 10,000 rpm for 5 min. Supernatant was placed in a clean tube and mixed with Proteinase K and AL Buffer and incubated at 70 °C for 10 min. Then 96% ethanol was added, samples were mixed, applied to spin columns and washed with AW1 and AW2 buffers. Purified DNA was eluted, measured with a NanoDrop 2000 spectrophotometer (ThermoScientific, Waltham, MA, USA) and stored at −20 °C until the analysis.

Bacteria (total bacteria, *Bacteroides* spp., *Lactobacillus* spp. and *Helicobacter* spp.) in fecal samples were evaluated by the amount of bacterial 16S rRNA DNA normalized to *Mus musculus* 28S rRNA DNA. PCR contained PCR Master Mix HS-qPCR SYBR Blue (Biolabmix, Novosibirisk, Russian Federation), specific primers with final concentration of 300 nM (all primer sequences are presented in Supplementary Materials Table S1), and 25–100 ng of DNA. Real-time PCR was performed in a CFX96 real-time PCR Detection System (BioRad Laboratories, Hercules, CA, USA) according to the following protocol: 95 °C for 3 min; 40 cycles of: 95 °C for 15 s, 62 °C for 25 s, 72 °C for 25 s; melt curve 65–95 °C. Relative amount of bacterial DNA was calculated by formula: 2^[Ct(28S rRNA)-Ct(16S rRNA)]^.

### 4.6. Sequencing of Tritrichomonas *sp.* DNA, Phylogenetic Analysis, and Tritrichomonas *sp.*, Clone Tsp1019 Real-Time PCR

To taxonomically determine the detected protozoa we performed Sanger sequencing of. 18S rRNA DNA in intestinal content of *Muc2^−/−^* mice using previously described primer sets [59]. Sequencing was performed using BigDye™ Terminator v3.1 Cycle Sequencing Kit (Applied Biosystems™, Warrington, Cheshire, UK) according to the manufacturer’s instructions. The reaction protocol was as follows: 96 °C for 60 s; 35 cycles of: 96 °C for 30 s, 56 °C for 15 s, 60 °C for 4 min. Then samples were purified by precipitation in 64% isopropanol (+4 °C) for 20 min at room temperature with following centrifugation at 14,000 rpm for 20 min. Invisible pallets were washed with 70% ethanol (+4 °C) and dried using vacuum concentrator. Samples were sequenced at the “Molecular and Cellular Biology” core facility of the IMCB SB RAS (Novosibirsk, Russian Federation).

Sequences obtained were analyzed using Unipro UGENE software [88], the assembly resulted in 1355 bp DNA sequence clone T.sp1019 (deposited in GenBank with accession number MT804340). The DNA sequence was analyzed using BLAST algorithm [89], for further analysis 11 sequences were picked (listed in Supplementary Materials Table S2). The sequences were aligned with MUSCLE algorithm using UGENE software and phylogenetic analysis was performed using IQ-tree web server [90] and visualized using FigTree software.

To further analyze the amount of this specific *Tritrichomonas* sp. Tsp1019 DNA in gut of mice we designed specific primers to the obtained sequence MT804340 (listed in Supplementary Materials Table S1), to verify unique specificity BLAST search was used. PCR analysis with fecal and colonic tissue DNA was performed as described above, according to the following protocol: 95 °C for 3 min; 40 cycles of: 95 °C for 15 s, 57 °C for 25 s, 72 °C for 25 s; melt curve 65–95 °C. Relative amount of protozoan DNA was calculated by formula: 2^[Ct(28S rRNA)-Ct(T.sp)]^.

### 4.7. RNA Extraction and Gene Expression Analysis

The gene expression analysis was performed according to our previously described protocol [58] with following modifications. Total RNA was purified from colon tissue samples using TRIzol reagent (Invitrogen, Waltham, MA, USA) and genomic DNA was removed by DNaseI (Roche, Mannheim, Germany) according to recommendations of the manufacturers. RNA concentration was determined using a NanoDrop 2000 spectrophotometer (ThermoScientific, Waltham, MA, USA). Reverse transcription reaction was performed using 7–10 µg of RNA, mix of random hexa-deoxyribonucleotide and Oligo-dT primers and M-MuLV reverse transcriptase (SibEnzyme, Novosibirsk, Russian Federation) according to the manufacturer’s recommendations. The cDNA obtained was five times diluted with deionized water and used for real-time PCR. Real-time PCR was performed using PCR Master Mix HS-qPCR SYBR Blue (Biolabmix, Novosibirisk, Russian Federation), 5 µL of cDNA, and 250 nM specific primers (listed in Supplementary Materials Table S3). The reaction was performed in a CFX96 real-time PCR Detection System (BioRad Laboratories, Hercules, CA, USA) according to the following protocol: 95 °C for 3 min; 40 cycles of: 95 °C for 15 s, 62 °C for 25 s, 72 °C for 25 s; melt curve 65–95 °C. Expression of the target gene was normalized to *Tubb5* level using the formula: 2^[Ct(Tubb5 mRNA)-Ct(gene of interest mRNA)]^.

### 4.8. Tritrichomonas *sp.* Microscopy

*Tritrichomonas* sp. microorganisms were harvested from cecum contents of mice using protocol previously described [64]. Briefly, cecum content was collected in 25 mL of ice-cold PBS, thoroughly resuspended, passed through a 70 µm cell-strainer and centrifuged at 1000 rpm for 5 min. A pallet was washed with ice-cold PBS, centrifuged as described above and resuspended in PBS. Then percoll 40/80% gradient was performed at 1000× *g* centrifugation for 15 min with breaks off and microorganisms were collected from the percoll 40/80 interface, centrifuged at 1750 rpm for 10 min and washed with ice-cold PBS. The microorganisms obtained were stained with Hoechst 33258 (Sigma-Aldrich, Darmstadt, Germany), mounted on a slide and examined under the fluorescent microscope (Zeiss Axio Imager M2, Carl Zeiss, Oberkochen, Germany) with ×100 magnification.

### 4.9. Histological Analysis

Colon tissue fixed in 4% paraformaldehyde were embedded in paraffin, *n* = 3 per group. Paraffin sections (4 µm) were stained with azur-II-eosin, the sections were examined in a blinded manner. Images were taken with an AxioImager.M2 microscope using an Axiocam 305 color camera (Zeiss, Oberkochen, Germany). Hyperplasia was defined as percentage of crypt elongation above the mean crypt length counted in C57BL/6 sections. Crypt length was determined using ImageJ software. Epithelial damage was defined as distortion of epithelial cell layer. PMN cells per section were counted on ×1000 magnification. 

Hyperplasia: 0: ≤10%, 1: 0–50%, 2: 50–100%, 3: ≥100%

Epithelial damage: 0—no pathological changes detectable, 1—epithelial desquamation, 2—erosion of the epithelial surface (gaps of 1 to 10 epithelial cells/lesion), 3—epithelial ulceration (gaps of > 10 epithelial cells)

PMN cell infiltration: 0: 1–30 per section, 1: 31–60 per section, 2: 61–90 per section, 3: ≥90 per section. 

### 4.10. Statistical Analysis

The data were tested for normality with the Kolmogorov–Smirnov test, all data had abnormal distributions. All data are presented as mean ± Standard Error of Mean (SEM), except for abundance of bacteria and Tsp1019 DNA, which are shown as actual values. The effect on body weight dynamics within the group was analyzed using Friedman test and differences between the groups were analyzed with Mann–Whitney u-test. All other data were analyzed using Kruskal–Wallis test and Mann–Whitney u-test. Within several data sets obtained for bacteria and Tsp1019 abundance some values were under detection limit, in that case Fisher Exact test was used.

## 5. Conclusions

Here we have demonstrated that the antibiotic treatment resulting in the bacterial microbiota depletion leads to dramatic weight loss and mortality in mice lacking mucin2 (*Muc2^−/−^* mice). This effect is associated with the increase of *Tritrichomonas* sp. in colon. Elimination of *Tritrichomonas* sp. using both an effective scheme of the antibiotic treatment and supplementation of the antibiotics with L-fucose, promotes survival of the mice. L-fucose does not affect viability and colonization of *Tritrichomonas* sp. infection in *Muc2^−/−^* mice with intact microbiota.

## Data Availability

The data presented in this study are available on request from the corresponding author.

## Supplementary Materials

The following are available online at https://www.mdpi.com/article/10.3390/ijms221910699/s1, Figure S1: Infection and breeding strategies being used to obtain *Muc2*^+/+^ and *Muc2*^−/−^ littermates infected with Tritrichomonas sp. (T.sp), Table S1: Primer sets used for microbial DNA PCR, Table S2: Sequences obtained by BLAST search and used for phylogenetic analysis, Table S3: Primer sets used for gene expression analysis.
