# Supplementary material for: Fucose Ameliorates Tritrichomonas sp.-Associated Illness in Antibiotic-Treated Muc2−/− Mice
Source: Int J Mol Sci. 2021 Oct 2;22(19):10699. doi: 10.3390/ijms221910699 (PMC8509520; doi:10.3390/ijms221910699)
Supplement: Supplementary file 1 [file ijms-22-10699-s001.zip › ijms-1401676-supplementary 1/Achasova et al_IJMS_supplementary files.pdf]

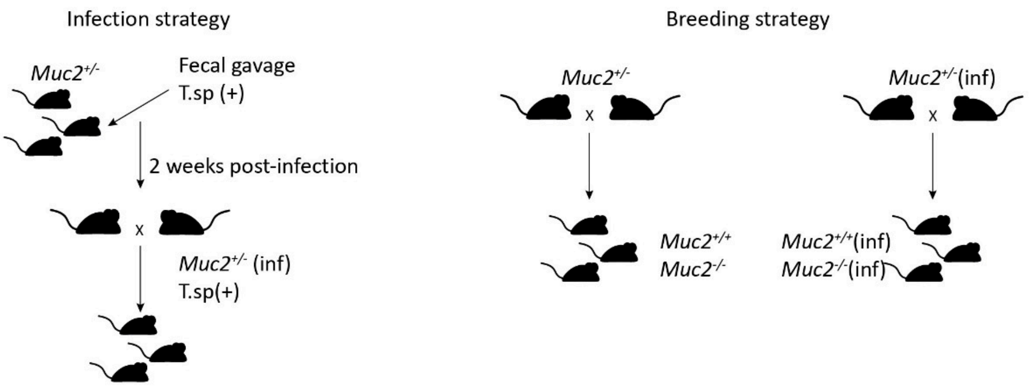

Figure S1. Infection and breeding strategies being used to obtain *Muc2*$^{+/+}$ and *Muc2*$^{-/-}$ littermates infected with *Tritrichomonas* sp. (T.sp).

Table S1. Primer sets used for microbial DNA PCR.

Primer name	5′->3′ sequence	Target	Annealing temp., °C
16Srrna-F	TCCTACGGGAGGCAGCAG	16S rRNA DNA, *Bacteria*	62
16Srrna-R	ATTACCGCGGCTGCTGG		
Bac.spp-F	GAGAGGAAGGTCCCCCAC	16S rRNA DNA, *Bacteroides* spp.	62
Bac.spp-F	CGCTACTTGGCTGGTTCAG		
Lac.spp-F	ATCTTCCACAATGG(G/A)CGC	16S rRNA DNA, *Lactobacillus* spp.	62
Lac.spp-R	GGCTGCTGGCACGTAGTTAG		
Hb.spp-F	CGGAGGGTGCAAGCGTTACT	16S rRNA DNA, *Helicobacter* spp.	62
Hb.spp-R	ATTCCACCTGCCTCTCCCAC		
Mus28S-F	CCTGGCGCTAAACCATTCGT	28S rRNA DNA, *Mus musculus*	62
Mus28S-R	AAAGCCCGCAGAGACAAACC		
T18S-F	GGAAGCACACTTCGGTCATAG	18S rRNA DNA	56
T18S-Ri	CCTTCCGTCAATTCCTTCAA		
T18S-Fi	AGGGTTTCTGTCGATCAAGG	18S rRNA DNA	56
T18S-R	CGTTACCTTGTTACGACTTCTCC		
T.sp-F	ATACCGCTTCCTGTTTTCA	*Tritrichomonas* sp., Tsp1019	57
T.sp-F	TTGGCAACTCTTTGATCCT		

Table S2. Sequences obtained by BLAST search and used for phylogenetic analysis.

Description	Percent identity	Accession number
Tritrichomonas sp. strain LL5	96.55%	MN120899.1
Tritrichomonas sp. MEG-2016a	96.42%	KX000921.1
Tritrichomonas muris clone 1-6	96.12%	AY886846.1
Tritrichomonas suis isolate L00998	96.02%	MK801504.1
Tritrichomonas foetus isolate NCSU Tfs-1	96.02%	AF466749.1
Tritrichomonas augusta strain LV2	96.02%	AY055802.1
Tritrichomonas nonconforma strain R114	95.80%	AY055803.1
Simplicimonas similis	91.06%	GQ254637.1
Monocercomonas colubrorum strain R293	90.52%	DQ174303.1
Joenia annectens clone KfJS4	90.10%	AB458854.1
Tetratrichomonas gallinarum strain KATASAMEC	88.36%	HQ149971.1

Table S3. Primer sets used for gene expression analysis.

Primer name	5′->3′ sequence	Target	Annealing temp., °C
Tubb5-F	TGAAGCCACAGGTGGCAAGTAT	Mouse *Tubb5* mRNA	62
Tubb5-R	CCAGACTGACCGAAAACGAAGT		
Tnf-F	CCCTCACACTCAGATCATCTTCT	Mouse *Tnf* mRNA	62
Tnf-R	GGCACCACTAGTTGGTTGTCTTT		
Ifng-F	CAAGTTTGAGGTCAACAACCCA	Mouse *Ifng* mRNA	63
Ifng-R	TCTTCCCCACCCCGAATCA		
Il1b-F	AAGGAGAACCAAGCAACGACAA	Mouse *Il1b* mRNA	63
Il1b-R	AACTCTGCAGACTCAAACTCCA		
Nos2-F	ATCGACCCGTCCACAGTATGT	Mouse *Nos2* mRNA	67
Nos2-R	CATGATGGACCCCAAGCAAGA		
